# Social context effects on error-related brain activity are dependent on interpersonal and achievement-related traits

**DOI:** 10.1038/s41598-018-38417-2

**Published:** 2019-02-11

**Authors:** José C. García Alanis, Travis E. Baker, Martin Peper, Mira-Lynn Chavanon

**Affiliations:** 1 Department of Psychology, Experimental and Biological Psychology, Neuropsychology Section, Philipps-Universität Marburg, Gutenbergstr. 18, 35032 Marburg, Germany; 2 Center for Molecular and Behavioral Neuroscience, Rutgers University, 197 University Avenue NJ, 0710 Newark, USA; 3 Department of Psychology, Child and Adolescent Psychology, Philipps-Universität Marburg, Gutenbergstr. 18, 35032 Marburg, Germany

## Abstract

Brain correlates of performance monitoring, such as the Error-Related Negativity (ERN), are considerably influenced by situational factors. For instance, errors committed during social interaction typically elicit enhanced ERNs. While individual differences in ERN magnitude have been implicated in a wide variety of psychopathologies, it remains unclear how individual dispositions may interact with situational incentives to influence performance monitoring. Here, we analysed how interpersonal (Affiliation) and achievement-related (Agency) traits moderated the effects of interpersonal competition and interpersonal cooperation on the ERN. For this purpose, electroencephalography was collected from 78 participants while they performed a Flanker Task either in a competitive or in a cooperative social context (i.e., between-subjects design). We found that competition predicted enhanced error-related activity patterns compared to cooperation. Furthermore, participants who scored high in Affiliation elicited enhanced error-related activity. Conversely, high Agency scores were associated with reduced error-related activity, but this was only observed in the competitive context. These results indicate that the brain’s response to error commission is not only sensitive to social incentives. Rather, the activity of the evaluative system that produces error signals appears to be crucially determined by the personal relevance of the incentives present in the context in which performance is evaluated.

## Introduction

In daily-life, committing an error can lead to undesired individual and social consequences. Accordingly, error commission triggers organismic and defensive reactions, such as heart rate deceleration^1^ and potentiated startle responses^2,3^, which are targeted to facilitate the error’s immediate detection and, if possible, behavioural adaptation. Empirical data suggest that individual differences in such physiological responses to error commission may account for individual variation in the degree to which errors induce subsequent behavioural adjustments^4^ and aversive emotional states^5,6^. Indeed, abnormal patterns of error-related physiological activity have been implicated in a wide variety of psychopathologies^7,8^. However, error processing is not only influenced by personal factors. Rather, error significance is also often depended on the context in which the error was committed. For instance, errors committed in the presence of others might be experienced as particularly distressing (cf.^9^). Here, we compared the influence of two social contexts on brain correlates of error monitoring and tested whether individuals’ interpersonal dispositions moderated these effects.

Research on human error processing has particularly focused on transient fluctuations in the scalp recorded electroencephalogram (EEG). Here, special attention has been paid to the Error-Related Negativity (ERN^10^ or Ne^11^; see^4,8,12^ for review). The ERN is a transient negative deflection of the Event-Related Potential (ERP) that peaks at fronto-central electrodes within 100 ms from error commission. The ERN is believed to reflect the activity of a performance monitoring neural network centred around the anterior cingulate cortex (ACC^13,14^). While the functional foundations of the ERN are still a matter of debate^12^, research indicates that the ERN reflects an endogenous alarm signal, which allows us to identify deviations from internally represented goals and adjust behaviour accordingly^15,16^. Further, these monitoring mechanisms show sensitivity to modulations of errors’ motivational salience, as indicated by selective enhancements of the ERN when particular emphasis is placed on behavioural accuracy^10^, or when errors lead to subsequent punishment^17–20^.

Overall, research on human error-monitoring has mainly focused on factors that impact the significance of individual behavioural performance, such as monetary gains or losses^17,21–23^. However, performance monitoring is also influenced considerably by social processes. For example, even in the absence of material losses, errors committed during social evaluation^18^, or in the mere presence of peers^24^, lead to similar enhancements of the ERN. It has been suggested that social contexts contribute to enhanced error processing by increasing the threatening value of errors for individuals’ self-esteem (e.g., by inducing concerns about being judged by others^25^). These findings are in line with a growing body of research associating enhanced ERNs with increased symptoms of social and more general forms of anxiety^6,26,27^, such as excessive concerns about poor behavioural performance (cf. checking behaviour^6^). Intriguingly, enhanced ERNs have also been linked to other interpersonal dispositions more closely related to positive affect, such as high sociability and agreeableness^21,28,29^. Although these findings may appear contradictory on first sight, they suggest that the functional basis of the ERN is closely related to how, or rather, how much individuals’ care about the consequences of their actions on a more general level. The manner by which these individual characteristics interact with situational factors (e.g., social interaction) to influence performance evaluation is still, however, poorly understood. The goal of the present study was to analyse how a context of social competition would modulate the amplitude of the ERN compared to a context of social cooperation. In addition, we analysed whether individuals’ interpersonal and achievement-related dispositions moderate these effects.

**Figure 1:**
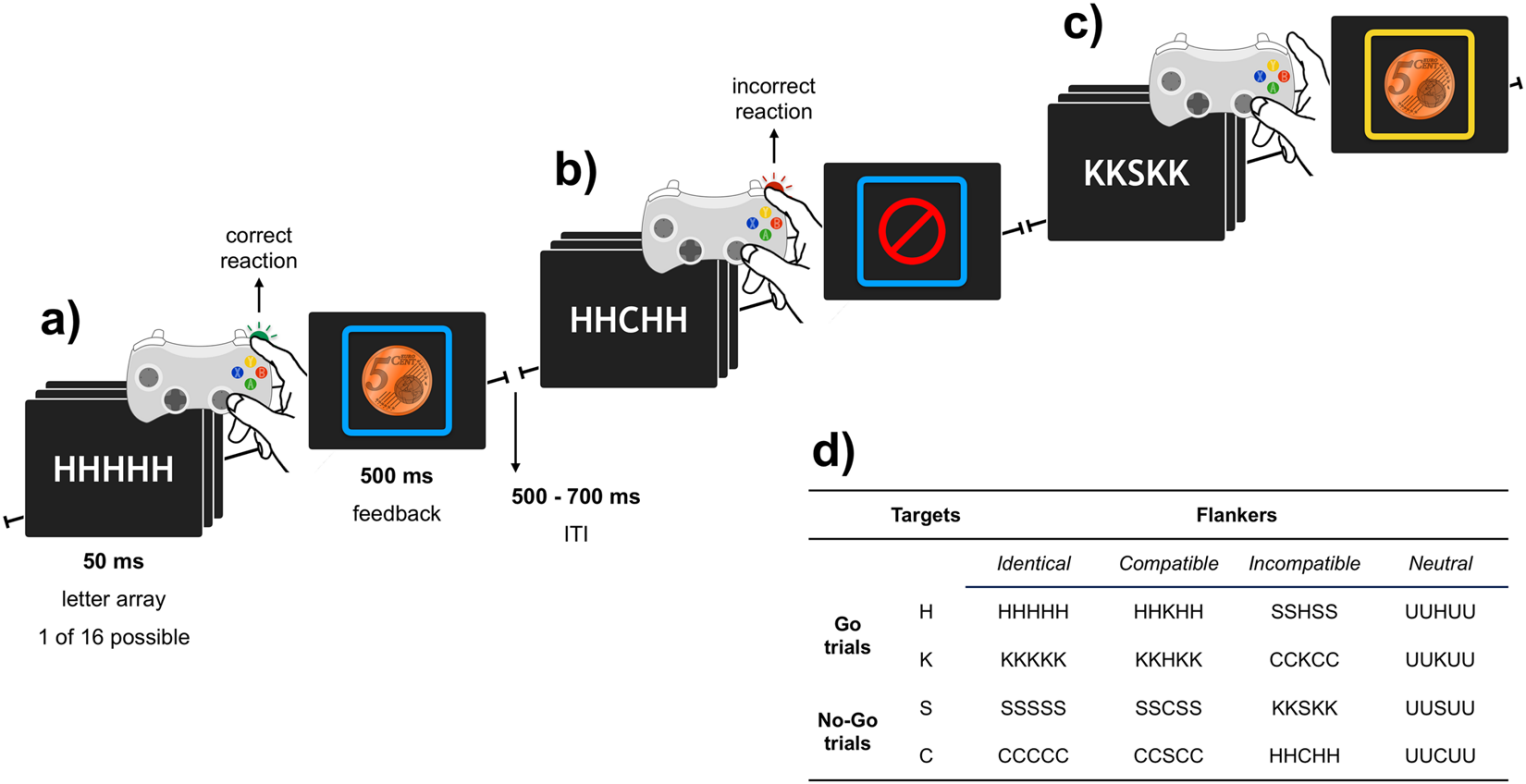
Schematic of the social flanker task. (**a**–**c**) Participants were presented with arrays of five-letters. There were 16 different letter combinations (see **d**). Participants were instructed to perform a target response when target letters were presented in the middle of the array. (**a**) Correct reactions were followed by feedback depicting a 0.05 € coin (1.4° diameter) and (**b**) errors were followed a red no-go sign (1.4° diameter). To differentiate between feedback relevant for the participant and feedback stimuli relevant for “the peer”, participant’s feedback was presented within a blue frame (see **a**,**b**). The peer’s feedback was presented within a yellow frame (see **c**).

For this purpose, participants were randomly assigned to one of two experimental groups. During the experiment, participants performed a social adaptation of the Eriksen Flanker task^30,31^ (see Fig. 1), either competing or cooperating with another person. In the competition group, participants were instructed to perform better than their opponent. In the cooperation group, participants needed to perform “well enough” as team to achieve reward. Unbeknownst to them, the second participant was a male confederate of the experimenter and his responses were simulated throughout the experiment. To test for the influence of personality on social error processing, we focused on two social subcomponents of interpersonal behaviour, which have often been related to the personality traits Extraversion and Agreeableness^32–34^. The first, Affiliation, relates to the experience of social warmth as well as the enjoyment and valuing of interpersonal bonds. The second, Agency, reflects a strong sense of achievement, assertiveness and the enjoyment of leadership roles. Indeed, previous research indicates that these subcomponents describe at least partially independent traits^35^.

Here, we hypothesise that interpersonal competition should highlight the significance of own behavioural performance (cf.^25^). Committing an error during social competition should induce an immediate negative comparison between self and others, leading to increased error salience, and thus enhanced (i.e., more negative) ERNs compared to ERNs elicited in a context of social cooperation (main effect of social context). Further, we postulate that personality moderates this effect. Affiliation describes a clearly interpersonal trait, which has been suggested to reflect individuals’ particular susceptibility to the motivational effects of social contexts^32–34^. Indeed, empirical data indicates that individual differences in the trait Affiliation may arise through the oxytocin-facilitated integration of salience and valence of reinforcers with social context information in the brain^34^. Thus, we expect individuals who score high in Affiliation to show increased error processing, reflected by enhanced ERNs compared to ERNs elicited by low Affiliation individuals. Intuitively, high Affiliation individuals should specially value social cooperation, as it delivers a context for establishing social relationships. As such, errors committed during social cooperation could endanger the relationship to the team-mate (i.e., errors disqualify oneself as a socially advantageous partner), increasing their motivational salience. Accordingly, high Affiliation individuals may show enhanced ERN amplitudes in the cooperation context compared to the competition context. Conversely, it is also possible that high Affiliation individuals elicit the strongest ERNs in the competition context. High in Affiliation individuals are characterised by a strong sensibility to social reinforcers and often tend to seek support from others in stressful situations^33,34,36^. Thus, high Affiliation individuals may experience errors as more motivationally salient in a context social competition, as, here, they are confronted with an opponent and not a supporting other. To the best of our knowledge, combined effects of Affiliation and social contexts have never been tested before on the ERN (although see^37,38^ for similar approaches on oxytocin). This makes it difficult to favour one of the previous hypotheses. Finally, the second trait, Agency, relates to a motivational pattern characterised by actively seeking and engaging in achievement related situations^32,33,39^. One of the key mechanisms that may predispose individuals with high Agency for their characteristic mastery-related and assertive motivational pattern may be a reduced sensitivity to failures, facilitating the anticipation of positive outcomes and supporting the maintenance of goal-directed behaviour in incentive contexts^40^. Thus, if high agency individuals are more sensitive to reward achievements than to failures, they should show reduced ERNs compared to low Agency individuals. In addition, if this effect depends on incentive motivation (cf.^40^), we expect it to be stronger in the competitive context.

## Results

For behavioural data analysis, we first compared participants’ error rates across trial types (i.e., describing the effect of flanker congruency; compatible, incompatible, identical and neutral) and across social contexts (i.e., competition vs. cooperation). Analyses were carried out using a 2-level mixed-effects regression approach (fitted by Maximum Likelihood), which estimated a random intercept for each participant. In addition, all models controlled for changes in participants’ degree of engagement in the task (∆Engagement; for details see Materials and Methods).

### Errors Rates

**Figure 2:**
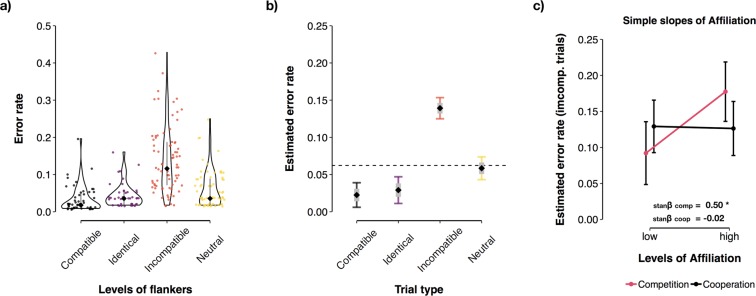
Results of the linear mixed-effects regression analysis of errors committed in the Flanker task. (**a**) Density, median, and interquartile range of the error rate in each of the trial types and (**b**) the corresponding estimates delivered by mixed-effects regression. The dashed line reflects the model intercept (mean estimated error rate) and black bullets depict the level estimate of error rate (grey bars = standard error, whiskers = 95% CI). (**c**) Effect of the personality facet Affiliation on the number of errors committed on incompatible trials. Error bars = 95% CI of the prediction. stanβ = standardised beta coefficient.

Participants’ error rates were significantly different across the levels of the factor Trial Type (F(3, 149) = 78.84, p = 2 × 10^−16^, _p_R^2^ = 0.613). As depicted in Fig. 2, incompatible trials were associated with a significantly increased error rate (M = 0.14, SD = 0.09) compared to compatible (M = 0.03, SD = 0.04; b_comp–incomp_ = −0.10, CI = −0.12–−0.08, p < 2 × 10^−16^), identical (M = 0.05, SD = 0.03; b_ident–incomp_ = −0.09, CI = −0.11–−0.07, p < 2 × 10^−16^) and neutral trials (M = 0.06, SD = 0.05; b_incomp–neutr_ = 0.06, CI = 0.04–0.08, p < 2 × 10^−16^). Identical trials and compatible trials were associated with significantly fewer errors compared to neutral trials (b_ident–neutr_ = −0.03, CI = −0.05–−0.01, p = 3 × 10^−03^; b_comp–neutr_ = −0.03, CI = −0.05–−0.02, p = 2 × 10^−05^). Conversely, the number of errors was not modulated by the factor Social Context (b_competition–cooperation_ < −0.01, CI = −0.02–0.02, p = 0.702), the interaction between Social Context and Trial Type (p = 0.760), or ∆Engagement (p = 0.393).

Based on these results, simple slopes analysis (cf.^41^) were conducted by fitting an ordinary least squares regression model on error data from incompatible trials, including the personality facets Affiliation and Agency as continuous predictors. We found a weak but significant interaction between the personality facet Affiliation and the factor Social Context (F(3, 149) = 4.22, p = 0.044, η^2^ = 0.015), indicating that, on average, participants who scored high in Affiliation and performed the task in the competitive context also committed a higher number of errors on incompatible trials (see Fig. 2c). Conversely there was no effect of the personality facet Agency (p = 0.370).

### Reaction Time

**Figure 3:**
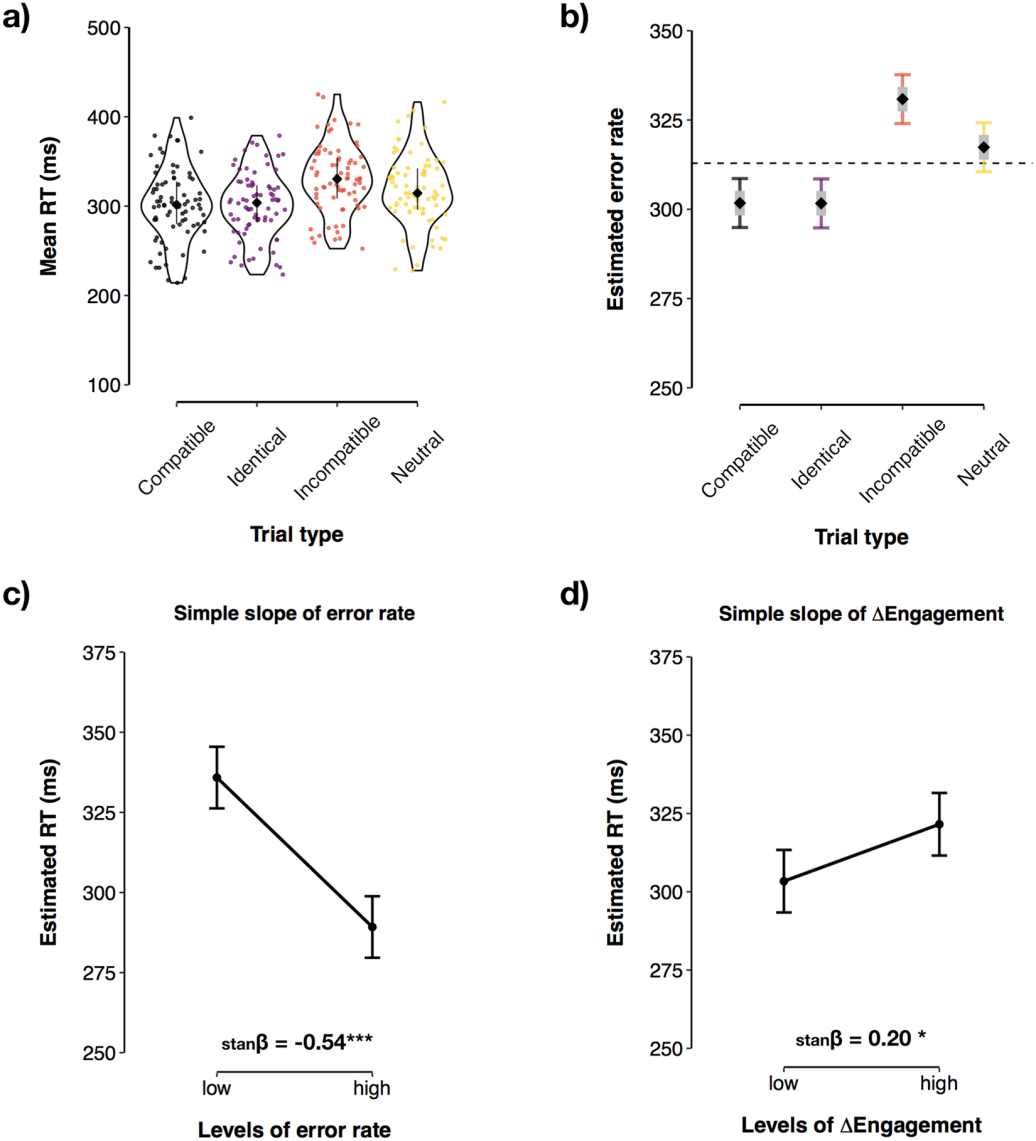
Results of the linear mixed-effects regression analysis of correct reactions’ reaction time. (**a**) Density, median, and interquartile range of mean reaction time (RT) in correct trials and (**b**) the corresponding mean RT estimates delivered by linear mixed-effects regression. Dashed line reflects the model intercept (estimated mean RT) and black bullets depict the level estimate of mean RT (grey bars = standard error, whiskers = 95% CI). (**c**) Effect of error rate on correct reactions’ RT and (**b**) effect of ∆Engagement on correct reactions’ RT. Dots depict predicted values. Error bars = 95% CI of the prediction. stanβ = standardised beta coefficient.

For analyses of reaction time, aggregated RT values (mean RT for each level of the factor Trial Type for each person) were modelled as a function of participants’ error rate, Social Context, the personality variables Affiliation and Agency, and participants ∆Engagement. These analyses indicated that correct reactions’ RT was not modulated by the factor Social Context (p = 0.447) or the personality facets Affiliation (p = 0.293) and Agency (p = 0.084). In contrast, RT was significantly different across the levels of the factor Trial Type (F(3,224) = 190.09, p < 2 × 10^−16^, _p_R^2^ = 0.459). As depicted in Fig. 3, compatible (M = 301.82 ms, SD = 40.77) and identical trials (M = 300.98 ms, SD = 36.58) displayed the fastest reaction times. Neutral trials (M = 318.04 ms, SD = 40.19) were significantly slower than identical (b_ident–neutr_ = −15.70 ms, CI = −19.57–−11.84, p < 2 × 10^−16^) and compatible trials (b_comp–neutr_ = −15.61 ms, CI = −19.49–−11.73, p < 2 × 10^−16^). Incompatible trials (M = 330.49 ms, SD = 38.43) had slower RTs than compatible (b_comp–incomp_ = −29.11, CI = −32.95–−25.27, p < 2 × 10^−16^), identical (b_ident–incomp_ = −29.21, CI = −33.05–−25.36, p < 2 × 10^−16^), and neutrals trials (b_incomp–neutr_ = 13.50, CI = 9.64–17.36, p =  < 2 × 10^−16^). Further, participants who committed less errors also displayed significantly slower RTs on correct trials (b = −466.29, CI = −605.19–−327.40, p =  < 2 × 10^−16^, _p_R^2^ = 0.370), indicative of individual differences regarding the extent to which individuals emphasised accuracy over speed (see Fig. 3c). Furthermore, we found a significant main effect of ∆Engagement (b = 4.54, CI = 0.81–8.28, p = 0.018, _p_R^2^ = 0.072): Participants with greater difference scores in ∆Engagement (i.e., participants who experienced a greater decline of task engagement throughout the task) displayed slower RTs on correct trials compared to participants with smaller ∆Engagement scores (see Fig. 3d).

### Error-Related Brain Activity

#### Context and personality effects on the amplitude of the ∆ERN

Physiological data was modelled in the same manner as behavioural data. First, brain activity specific to error commission (i.e., the ∆ERN) was computed by subtracting the scalp-recorded physiological response elicited by correct reactions (i.e., the CRN) from the physiological response elicited by errors (i.e., the ERN). ∆ERN amplitude recorded 0 to 100 ms after motor response at the electrodes FCz and Cz (for details see Methods Section and Supplementary Fig. S1) was then modelled as a function of the factor Social Context and the personality facets Affiliation and Agency. Further, all models controlled for incompatible trials’ error-rate and ∆Engagement.

**Figure 4:**
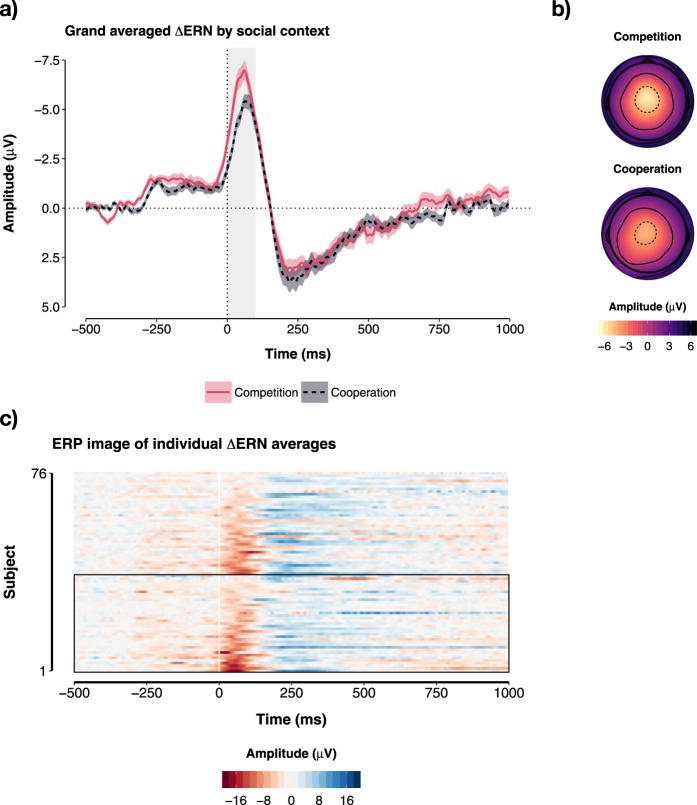
∆ERN by social context. (**a**) Time course of the ∆ERN (pooled from the electrodes FCz and Cz) for each social context. (**b**) Topographical maps for mean activity recorded from 0 to 100 ms after motor response. The shaded regions surrounding the lines in (**a**) reflect standard errors. (**c**) ERP Image of the ∆ERN depicting each subject in the experiment. Subjects wo performed the task in the competitive social context are shown within the box. Subjects were sorted according to ∆ERN magnitude.

In line with our predictions, the amplitude of the ∆ERN (i.e., the differential activity between errors and correct reactions) was significantly different across the levels of the factor Social Context (b_competition–cooperation_ = −1.40, CI = −2.63–−0.16, p = 0.027, _p_R^2^ = 0.062). This indicated that, on average, individuals who performed the task in the competitive context generated more pronounced ∆ERNs (M = −5.68 µV, SD = 4.21) compared to individuals who performed the task in a cooperative context (M = −4.27 µV, SD = 3.22; see Fig. 4).

**Figure 5:**
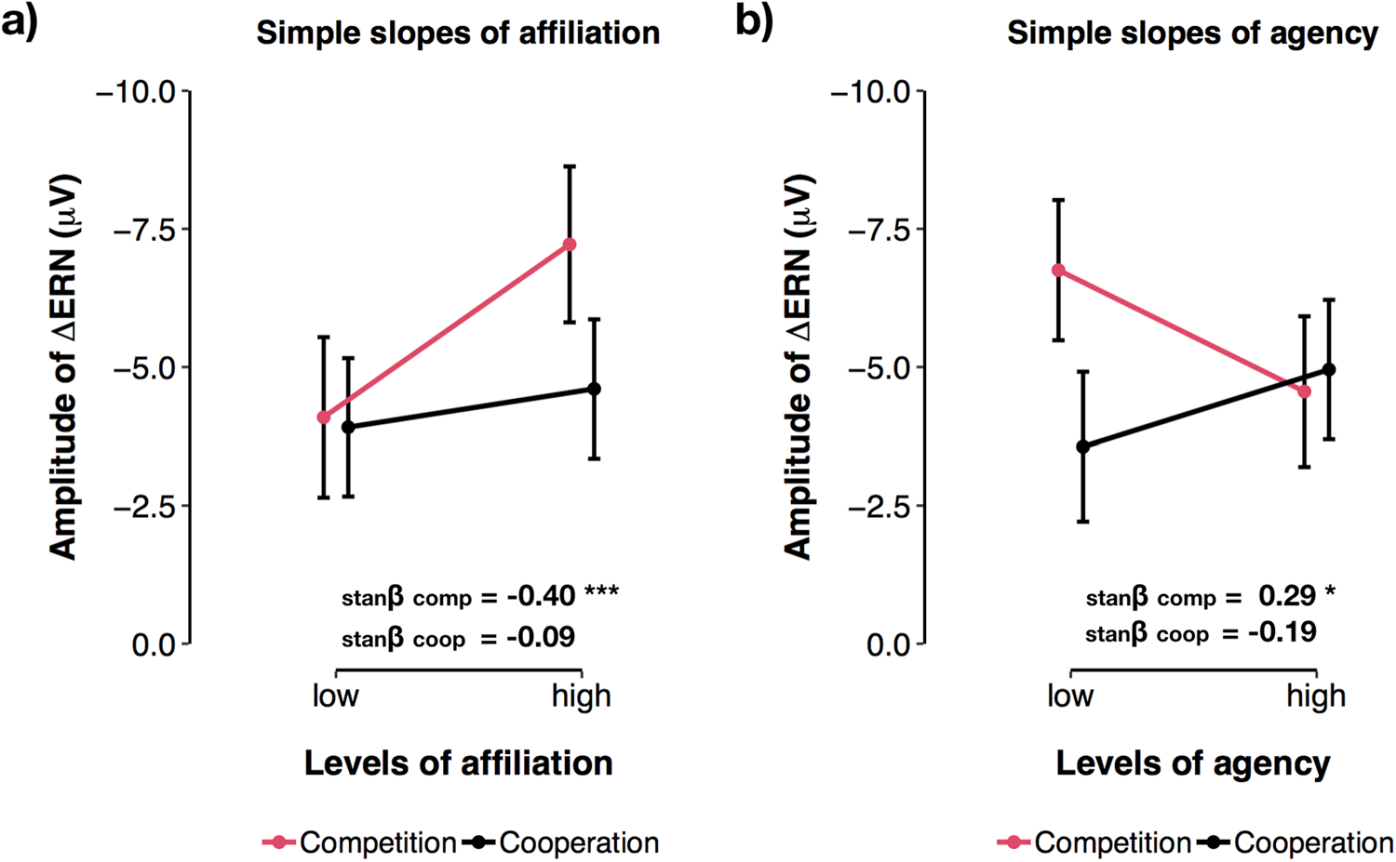
Personality effects on the amplitude of the ∆ERN. (**a**) Affiliation × social context interaction. (**b**) Agency × social context interaction. Error bars = 95% CI of the prediction. stanβ = standardised beta coefficient.

In addition, we found a significant main effect of Affiliation on ∆ERN amplitude (b = −0.24, CI = −0.42–−0.06, p = 0.010, _p_R^2^ = 0.083). This indicated that, participants who scored high in Affiliation also elicited more pronounced ∆ERNs. Simple slopes indicated that this effect was strongest in the competitive context (b_competition_ = −0.39, CI = −0.67–−0.11, p = 0.007; b_cooperation_ = −0.09, CI = −0.31–0.14, p = 0.450; see Fig. 5a). There was no significant main effect of Agency on the ∆ERN (p = 0.565). However, we found a significant two-way interaction between Agency and the factor Social Context (F(1, 76) = 6.66, p = 0.011, _p_R^2^ = 0.081). Simple slopes analyses indicated that, in the competitive context, high Agency scores were associated with significantly less pronounced ∆ERNs (b = 0.08, CI = 0.01–0.14, p = 0.027), while this effect was reversed in the cooperative context (b = −0.05, CI = −0.12–0.02, p = 0.163; see Fig. 5b). We found no significant main effect of ∆Engagement (p = 0.085) or error rate (p = 0.164) on the amplitude of the ∆ERN.

#### Context and personality effects on the amplitude of the ERN and the CRN

To better understand these effects, follow-up analyses were carried out by re-fitting the model. However, this time Reaction (error vs. correct) was included as a within-subjects factor. Furthermore, the model estimated a random slope of Reaction for each participant.

**Figure 6:**
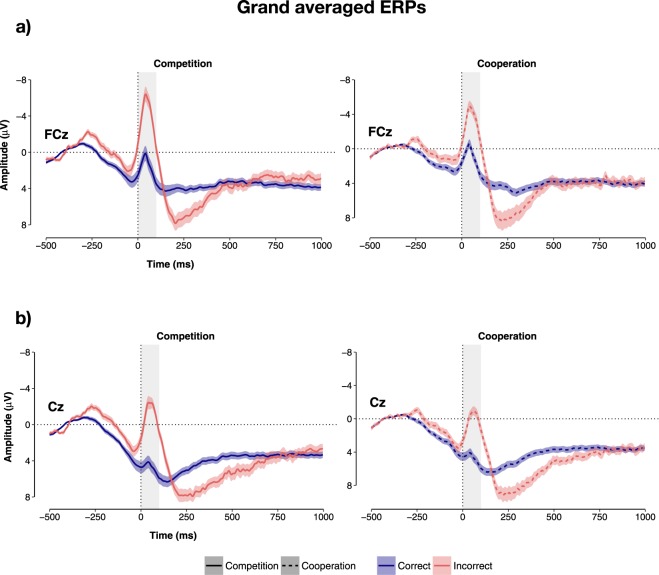
Grand averaged ERN and CRN by social context. Time course of the ERN and CRN for each social context at the electrodes (**a**) FCz and (**b**) Cz. Shaded regions surrounding the lines in reflect standard errors.

These analyses revealed that, overall (i.e., regardless of behavioural response), amplitude values were not affected by the factor Social Context (p = 0.322), Affiliation (p = 0.478) or Agency (p = 0.588). In contrast, there was a significant main effect of the factor Reaction (b_error–correct_ = −4.96, CI = −5.57–−4.34, p < 2 × 10^−16^, _p_R^2^ = 0.774; see Fig. 6) indicating that, on average, the physiological response recorded during errors (i.e., the ERN) was significantly stronger (i.e., more negative; M = −1.97 µV, SD = 4.49) than the physiological response recorded during correct reactions (i.e., the CRN; M = 2.99 µV, SD = 4.27).

In addition, we found a significant two-way interaction between the factor Reaction and the factor Social Context (F(1, 76) = 5.63, p = 0.020, _p_R^2^ = 0.069). Mirroring the effects described above, the estimated differential amplitude between ERN and CRN was stronger in the competitive context (b = −5.69, CI = −6.56–−4.80, p < 2 × 10^−16^) compared to the cooperative context (b = −4.23, CI = −5.08–−3.37, p < 2 × 10^−16^). Simple slopes indicated that while the amplitude of the CRN was comparable in both contexts (M_comp_ = 3.17 µV, SD = 4.73; M_coop_ = 2.82, µV, SD = 3.79; b_competition–cooperation_ = 0.10, CI = −1.30–1.50, p = 0.890), there was a trend for stronger ERN amplitudes in the competitive context (M = −2.51 µV, SD = 4.63) compared to the cooperative context (M = −1.45 µV, SD = 4.29; b_competition–cooperation_ = −1.36, CI = −2.76–0.04, p = 0.056).

**Figure 7:**
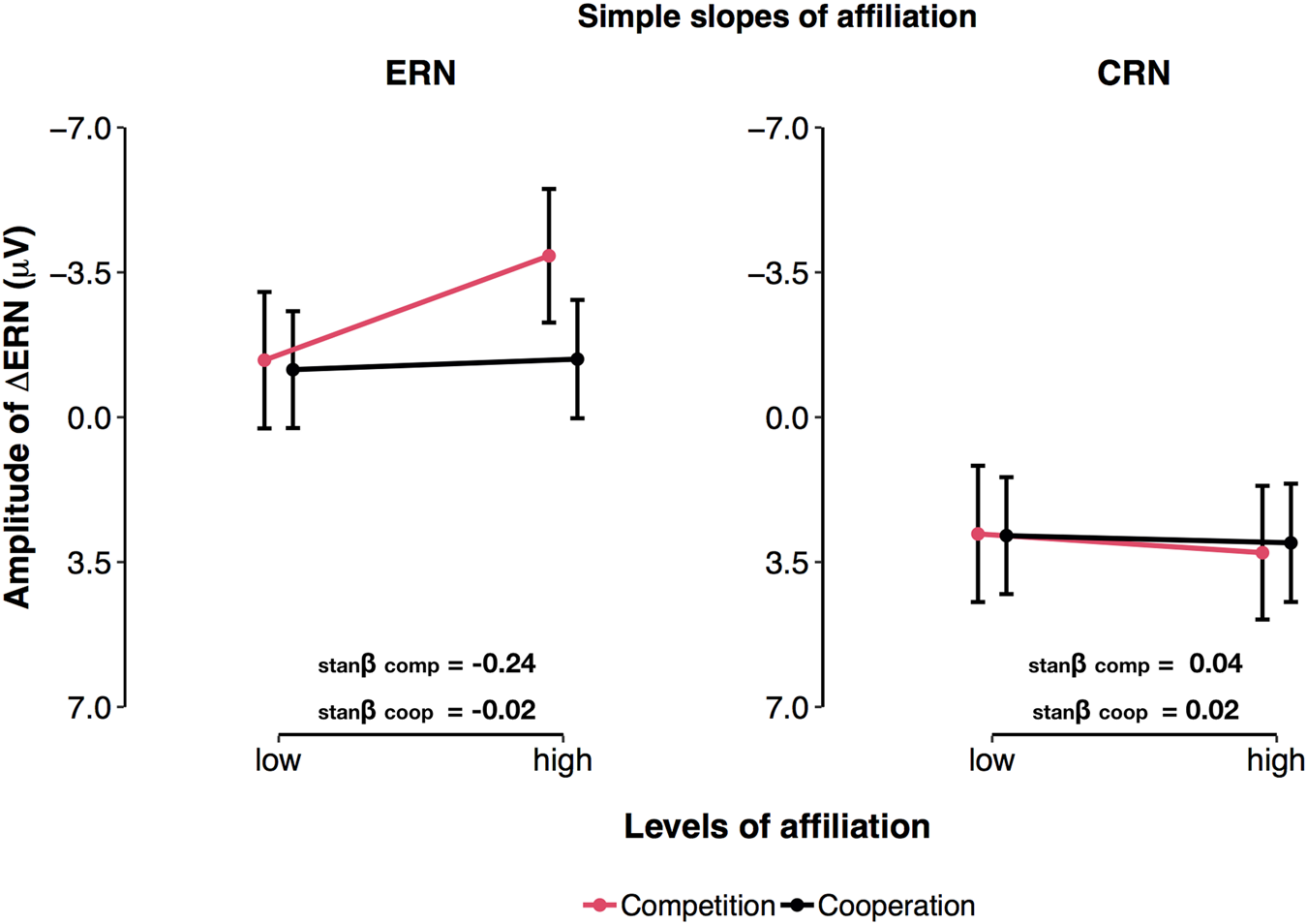
Affiliation effects on the amplitude of the ERN and CRN. (**a**) Effects of Affiliation × Social Context interaction on the ERN. (**b**) Effects of Affiliation × Social Context interaction on the CRN. Error bars = 95% CI of the prediction. stanβ = standardised beta coefficient.

Further, a significant interaction between Affiliation and the factor Reaction was observed (F(1, 76) = 5.58, p = 0.021, _p_R^2^ = 0.684). This interaction corresponds to the main effect of Affiliation on the ∆ERN described above: The difference between ERN and CRN was more pronounced in individuals with high Affiliation scores (i.e., +1 SD; b = −5.81, CI = −6.75–−4.87, p < 2 × 10^−16^), compared to individuals with low Affiliation scores (i.e., −1SD; b = −4.10, CI = −5.05–−3.15, p < 2 × 10^−16^). Simple slopes indicated that this effect emerged because high scores in Affiliation were associated with descriptively enhanced ERNs (b_low–high affiliation_ = 1.39, CI = −0.26–3.04, p = 0.099), while Affiliation had no substantial effect on the amplitude of the CRN (b_low–high affiliation_ = 0.31, CI = −1.97–1.34, p = 0.708; see Fig. 7).

**Figure 8:**
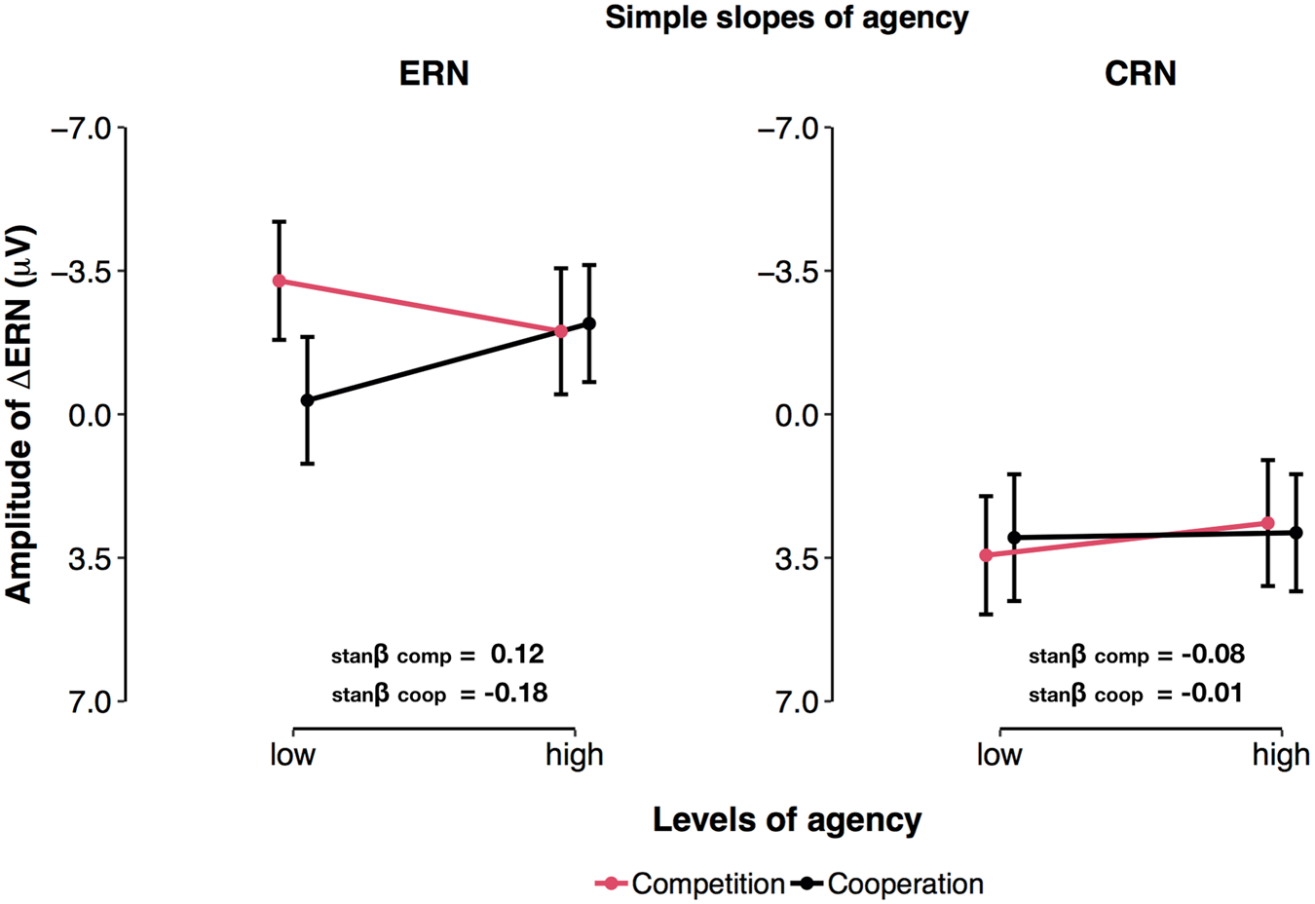
Agency effects on the amplitude of the ERN and CRN. (**a**) Effects of Agency × Social Context interaction on the ERN. (**b**) Effects of Agency × Social Context interaction on the CRN. Error bars = 95% CI of the prediction. stanβ = standardised beta coefficient.

Finally, we found a significant three-way interaction between Agency, the factor Reaction, and the factor Social Context (F(1, 76) = 7.46, p = 0.008, _p_R^2^ = 0.089). This interaction corresponds to the differential effect of Agency on the ∆ERN described above. In the competitive context, the difference between ERN and CRN was more pronounced in individuals with low Agency (i.e., −1SD; b = −6.70, CI = −7.96v5.43, p = 1 × 10^−16^), compared to individuals with high Agency (i.e., +1 SD; b = −4.68, CI = −6.03–−3.33, p = 1 × 10^−09^). In contrast, in the cooperative context, the difference between ERN and CRN was more pronounced in individuals with high Agency (i.e., +1 SD; b = −5.10, CI = −6.35v3.85, p = 6 × 10^−12^), compared to individuals with low Agency (i.e., −1SD; b = −3.35, CI = −4.69–−2.00, p = 4 × 10^−06^). As depicted in Fig. 8, simple slopes indicated that these effects emerged because, high Agency scores were associated with descriptively less pronounced ERNs (b_low–high agency_ = −1.23, CI = −3.44–0.98, p = 0.271) and enhanced CRNs (b_low–high agency_ = 0.79, CI = −1.42–2.99, p = 0.482) in the competitive context. Conversely, in the cooperative context, high Agency was related to descriptively enhanced ERNs (b_low–high agency_ = −1.87, CI = −0.37–4.12, p = 0.101), but did not influence the CRN (b_low–high agency_ = 0.12, CI = −2.13–2.37, p = 0.916).

## Discussion

In line with our predictions, participants who performed the Flanker task in a context of interpersonal competition elicited enhanced error-related brain activity. This corresponds with previous empirical data indicating that the magnitude of error-related brain signals varies in accordance to factors that affect the motivational significance of behavioural performance. For instance, ERN amplitudes are typically enhanced when errors are punished with monetary losses^17,19^, or when errors are followed by a loud and aversive sound^18^. Our results extend these findings by indicating that social contexts may differentially influence individuals’ physiological response specific to error commission, the ∆ERN, and that this effect arises from a modulation of the underlying ERN. Importantly, these effects were independent of changes in participants’ degree of engagement and performance in the task. Similar to previous studies (cf.^25,42^), we did not find a direct behavioural equivalent for the physiological effects reported here (but see Supplementary Materials for social context effects on post-error slowing). However, the absence of social context influences on behavioural task performance also indicates that the differential error-related activity patterns elicited in the competitive and cooperative contexts were not explained by general differences in the numbers of trials used for ERP analyses.

One possible explanation for enhanced ∆ERNs in the competitive context is that, here, errors were experience as more motivationally salient. For instance, in the competitive context, goal achievement was predominantly dependent on participants’ self-generated actions. Thus, enhanced ∆ERNs may reflect a stronger orienting response to ongoing deviations from goal-directed behaviour. In contrast, in the cooperative context goal-achievement was not entirely dependent on participants’ own performance, as, in principle, errors could be compensated by the peer. Enhanced ∆ERNs during interpersonal competition could also be facilitated by evaluation apprehension processes^25^. In the competitive context, participants were told that only the subject with the lowest error rate would be rewarded at the end of the task. Thus, it is possible that each error committed during competition implicated an immediate negative comparison of one’s own performance to that of the peer’s, activating concerns about social devaluation^25^ and increasing the motivational significance of ongoing error commission. Indeed, making a mistake in a context that explicitly implies social comparison can interfere with a desire to appear accurate and confident as we strive to achieve goals^42^. Accordingly, one could also expect a similar effect in the cooperative social context, as here, committing an error could lead to collective failure and feelings of embarrassment. However, we observed blunted ∆ERNs in cooperation compared to competition. This could be an epiphenomenon of our experimental procedure. In the cooperation context, participants were told that they would be rewarded at the end of the task if their collective error rate was good enough. Further, participants learned during the task that a feedback about individual contributions to goal achievement was not provided. Therefore, concerns about social devaluation might be less prominent in the cooperation context implemented here.

Accordingly, individuals who scored high in Affiliation, a personality trait believed to reflect individual’s susceptibility to social settings and valuing of interpersonal interaction^32–34^ elicited the most pronounced ∆ERNs. Furthermore, this effect was descriptively stronger in the competitive context. Importantly, the effect of Affiliation was independent of changes in participants’ degree of engagement during the task. Rather, we suggest that the negative relation between error-related brain activity and Affiliation indicates that failing in a context that explicitly implies social comparison induces an enhanced subjective experience of threat in socially motivated individuals (cf. sustained threat^6^). Indeed, in a recent study by Barker and Colleagues^42^, social evaluation enhanced the ∆ERN in socially anxious individuals but not in healthy controls (see^43^ for similar age-related effects). Moreover, the magnitude of this enhancement was significantly correlated with the severity of social anxiety symptoms^42^. As affiliation-related traits have been previously reported to show weak but negative correlations with social anxiety symptoms^44^, these effects might appear contradictory. Interestingly however, paradoxical interaction effects between Affiliation inducing drugs (e.g., Oxytocin) and elevated levels of anxiety have been reported before^38,45^. For instance, Oxytocin administration may actually facilitate stress and anxiety, when tasks are performed in a hostile environment^46,47^. In addition, elevated levels of endogenous Oxytocin, a putative correlate of high Affiliation^34^, have also been shown to correlate with increased experience of interpersonal distress and increased symptoms of social anxiety^48,49^. Together with these findings, our results suggest that Affiliation and social anxiety might share a common motivational pattern. For instance, social anxiety is not only characterised by fear of social rejection. Rather, social anxious individuals often report normal or enhanced levels of desire to be with others^50,51^. Thus, a possible explanation for enhanced error-related activity in high Affiliation individuals and social anxiety could be that both populations regard social interaction and social ties as particularly relevant. Furthermore, social settings might represent situations where both groups engage in enhanced performance monitoring and experience errors as more threatening. Combined with the effects induced by the competitive social context, these results indicate that individual differences in the ∆ERN could represent a valuable tool for understanding how and to what degree individuals experience social incentives. For instance, whether individuals are actively, or even excessively, concerned about the consequences of their behaviour in social settings^6,52^.

In the present study, ∆ERN amplitude was also significantly influenced by participants’ scores in Agency, a personality trait believed to reflect high achievement motivation and an assertive and dominant social interaction style (cf.^35^). Here, individuals who scored high in Agency showed significantly reduced error-related activity in the competitive context. This finding suggests that, in potentially rewarding environments, individuals with high Agency elicit a reduced orienting response to error commission. One possible explanation is that, in incentive settings, individuals with high Agency may rely more on positive incentives (e.g., potential rewards) to maintain reward-seeking behaviour and endure failure. This is in line with previous reports suggesting that Agency-related personality traits arise from a reduced sensitivity to signals of punishment compared to signals of reward^39,40^. For instance, Mueller and Colleagues^40^ reported a similar effect of Agency on frontal midline activity in the theta frequency band upon presentation of negative performance feedback. These frequency responses to negative feedback are believed to represent the functional counterpart of the ERN, reflecting transient failure-processing when outcomes are worse than expected (cf.^15^). In Mueller’s study^40^, high Agency was associated with decreased theta power responses to negative feedback only after potential rewards were introduced and presented to the participants. In the absence of potential rewards, theta power responses to negative feedback displayed no relationship with Agency, while they showed a positive correlation with measures of anxiety (i.e., neuroticism). Thus, the findings by Mueller and colleagues and the present results make a case for contextualised personality effects on error-related brain activity and highlight that these processes crucially dependent on specific stimuli and situations that activate underlying motivational patterns.

Together, our results suggest that the link between the evaluative system that produces performance monitoring ERPs and factors that influence the motivational significance of behaviour (e.g., signals of potential rewards and/or social evaluation) is crucially determined by how individuals appraise the relevance of the incentives present in the context in which performance is evaluated. For instance, whether individuals describe themselves as wanting or liking these incentives (cf.^53^), where the former refers to whether a stimulus induces a strong approach-motivation and the later refers solely to the enjoyment of the stimulus itself. This is particularly interesting in the light of the Research Domain Criteria (cf.^8^), a framework that intends to identify neural and biological markers of psychiatric disorders. Here, a growing body of research supports the existence of a reliable link between enhanced error-related brain activity and social (cf.^54^) and more general anxiety disorders (cf.^55^). Our results extend these findings by indicating that individual differences might be more reliable when induced by relevant stimuli and situational settings. For instance, our results indicate that, similar to socially anxious persons, individual who describe themselves as valuing interpersonal contact on a more general level also show enhanced error-related brain activity when errors are committed in the context that explicitly implies social evaluation. This suggests that the ERN is more closely related to specific concerns experienced by individuals in specific situational settings, rather than general anxiety (cf.^6^). Accordingly, we found that Agency reduced the magnitude of this response. Agency-related traits^56^ and reduced error-related brain activity^57^ have been repeatedly discussed in terms of anomalies in mesocorticolimbic dopamine function, suggesting that the key mechanism driving these effects is a stronger orienting response to signals of reward relative than to signals of punishment (cf.^39,40^). Indeed, reward-related activity has also been reported to show some degree of dependency on personal and situational relevance. For instance, in substance dependent individuals, drug-related rewards (e.g., cigarette puffs) elicit enhanced reward-related brain activity compared to monetary rewards during the state of drug craving (e.g., nicotine deprived smokers^58^).

Certainly, our results require further replication. Future research should improve some of the design characteristics implemented here. For example, participants only performed the task in one of two social contexts. It is therefore unclear whether individuals who elicited a strong error-related response in one context would elicit comparable responses in the other context. Thus, a complete dissociation of the unique contributions of personality and social contexts remains difficult. Further, it appears plausible that social cooperation should also enhance the relevance of own behavioural performance, particularly in socially motivated individuals. However, we did not analyse individuals’ error-related response in the absence of social interaction. Further research should incorporate a neutral condition in which participant may perform the task on their own. In addition, a comparison to other forms of incentive motivation (e.g., monetary vs. social rewards) could help isolate the effects of interpersonal and achievement-related motivation. On the other hand, our sample was limited to male undergraduates. Previous research suggest that gender influences the physiological response to error commission^59^ as well as the propensity for concerns about poor social performance considerably^60^. Thus, further research including female participants is necessary to elucidate the interaction between social contexts, Affiliation and Agency related traits.

In conclusion, we found supporting evidence for the role of the ERN as an endogenous attention-call signal elicited by error commission. Additionally, we were able to extend previous findings indicating that personal (cf.^37,42^) and situational factors (cf.^25^) that contribute to the salience of social incentives lead to an enhancement of this orienting response. We suggest that the interaction between these factors is crucial to understand how individuals appraise the motivational significance of performance monitoring. Furthermore, our results could provide novel directions for the development of clinical trials regarding a variety of disorders, such as social anxiety disorder and substance abuse. A challenge for further research relies in the analysis of how contextualised dispositions, i.e., situation-dependent personality effects, may help design interventions to ameliorate or prevent maladaptive patterns of performance evaluation.

## Materials and Methods

### Participants

Seventy-eight right-handed males were included in the study. Only males were recruited to avoid interactions with the confederate’s sex^61^. Exclusion criteria were acute and/or history of psychiatric and neurological disorders, as well as current use of prescription drugs. All participants had normal or corrected to normal vision and provided written informed consent prior to participation. Participants received course credit for participation and a monetary reward according to their performance in the task (i.e., up to $18). Two subjects were removed from analysis due to technical issues during EEG recording. Thus, the final sample consisted of 76 subjects (37 in the competition context and 39 in the cooperation context, M_age_ = 23.32, SD = 2.99). The present study was approved by the Department of Psychology’s ethics committee at the Phillips-University of Marburg and all procedures carried out in accordance with the guidelines.

### Procedure and experimental task

As a cover story, subjects were recruited to participate in a study aimed to identify brain correlates of performance monitoring in “two-player tasks”. Subjects agreed to be the EEG-participant during recruitment. Upon arrival to the lab, subjects filled out personality questionnaires, provided general demographic data and were prepared for EEG recordings. Importantly, prior to the experimental task, participants were briefly introduced to the second participant, “the peer”, a male confederate of the experimenter. Participants were able to see the peer through an open door as the experimenter provided them with general instructions. In order to enhance the authenticity of the paradigm, the participant and the peer were encouraged to wait for each other before proceeding through the instruction slides presented on their computer screen. After a short practice session, participants performed a social go/no–go adaptation of the Eriksen Flanker Task^30^ (performed using Presentation® software, Neurobehavioral Systems, Inc., Berkeley, CA). Hereafter, there was no further interaction with the peer or the experimenter.

The social flanker task consisted of 672 flanker trials separated by short self-paced pauses every 84 trials. On each trial, participants were presented with one of 16 different five-letter arrays (50 ms; width 2.9°, height 0.6°, white letters on black background; see Fig. 1). Participants were instructed to use the index finger of the right hand to respond to the letters H and K (i.e., the targets) when these were presented in the middle of the letter array (i.e., go-trials). Conversely, participants were told that the peer’s targets consisted of the letters S and C. Further, participants were instructed to withhold their reaction when the peer’s targets appeared in the middle of the array (i.e., no-go trials; 50% of all trials). Targets letters were surrounded by distracting letters (i.e., flankers). These could be either identical to the target (i.e., identical trials; e.g., HHHHH), compatible, as they also referred to go behaviour (i.e., compatible trials; e.g., KKHKK), incompatible, as they were associated with no-go behaviour (i.e., incompatible trials; e.g., SSHSS), or neutral, as they didn’t refer to any behavioural tendency (i.e., neutral trials; e.g., UUHUU). Letter arrays were followed by a short response window (800 ms). Subsequently, performance feedback was presented for 500 ms. Inter-trial interval varied between 500–700 ms.

### Manipulation of social task context

The competitive or cooperative context of the task was determined after the practice session. For this purpose, the participant’s recruitment code was matched to a previously randomised code list. In both task contexts, participants saw instruction slides indicating that correct responses would be rewarded with 0,05 €. In the cooperative context, participants were told that their gains would only be disbursed if they managed to achieve a less than 10% error rate as a team, and that each subject would receive half of the accumulated sum. Thus, if the participant committed 5% errors, while the peer reached a 10% error rate, their mean error rate would be 7.5% and their collective gain would be disbursed. In contrast, in the competitive context, participants were instructed that of each dyad only the participant with the lowest error rate would be rewarded at the end of the experiment. To make sure that in both contexts, payoffs were predominantly dependent on the participant’s performance and task demands were as comparable as possible, the performance of the peer was virtually simulated. It consisted of a random distribution 95% correct and 5% missed reactions in “peer go-trials” (i.e., participant no-go trials). Further, as the peer could only commit errors in trials that entailed the actual participant’s targets (and vice versa), the task was programmed to display simulated feedback indicating that the peer had committed an error in 10% of all participant’s correct responses. In other words, participants saw the peers no-reward feedback even though they had pressed the correct button in 10% of the cases. This way the peer’s error rate was kept constant at roughly 10%, regardless of context.

To control for the effects of general task engagement, participants were asked to rate their current mood with help of two bipolar items (e.g., 1 = not motivated at all vs. 9 = very motivated; 1 = indifferent vs. 9 = interested). The items were presented at the beginning and at the end of the task. Mean scores were used to create an overall measure of momentary task engagement (Cronbach’s alpha = 0.70 − 0.82). Subsequently, scores at the end of the task were subtracted from the scores at the beginning of the task to create an engagement difference score (∆Engagement). Thus, higher ∆Engagement scores indicate a stronger decreased of task engagement throughout the task. This score was used as a continuous covariate in further analyses.

### Affiliation and agency

Affiliation was assessed using the Social Closeness Scale of Tellegen and Wallers’ Multidimensional Personality Questionnaire^62^. It consists of 21 self-report items designed to measure individuals’ sociability. Individuals with high scores in the Social Closeness Scale describe themselves as liking to be with other people, taking pleasure in and valuing close interpersonal ties^62^. The Social Closeness Scale has been reported to show a strong internal consistency^35^ (Cronbach’s alpha ~ 0.82–0.89). In the current sample internal consistency was 0.78.

Agency was assessed using the Marburg Agentic Extraversion Scale (MAE^63^). It comprises three positively correlated 10-item scales that match the primary trait-scales of the Tellegen and Wallers’ MPQ^62^: Social Potency, Achievement, and Wellbeing (cf.^64^). The sum of these three scales constitutes an aggregated measure of agency^63^. Individuals with high scores in agency describe themselves as assertive and decisive (Social Potency), hard-working and having high performance standards (Achievement), being generally optimistic and feeling good about themselves (Wellbeing). In line with previous reports^63^ the internal consistency of the MAE- was 0.89 in the current sample. In our study, Agency and Affiliation displayed a medium size correlation (r = 0.35, p = 0.03).

### EEG-data acquisition and processing

Participants seated in a light-attenuated room while EEG data was recorded continuously from 64 silver/silver-chloride active scalp electrodes placed according to the 10–20 System^65^. Data were digitalised at a sampling rate of 512 Hz (2048 Hz at ¼ decimation rate) using ActiView software (Version 6.05, BioSemi, Amsterdam, NDL). Electrodes on the supra- and infraorbital rims as well as on outer canthi of the eyes recorded eye movements (electrooculogram). Online, data was referenced to two linked electrodes located midway between POz and PO3/PO4 respectively (Common Mode Sense active electrode and Driven Right Leg passive electrode; all electrode offsets were kept below 25 mV).

Offline, data were pre-processed using EEGLAB Version 13.6.5b^66^ running on MATLAB 2016b (The MathWorks Inc., Natick, MA). Here, data were sampled down to 256 Hz and re-referenced to the average of all 64 electrodes. Data were bandpass filtered using a Hamming window finite impulse response filter (lower cut-off = 0.25 Hz; upper cut-off = 50.25, order = 1690). Next, EEG data were individually decomposed using Adaptive Mixture Independent Component Analysis (AMICA^67,68^). Components representing stereotypical artefacts, such as ocular movements, muscular activity and noise were removed and the remaining components back-projected (see Supplementary Materials). The pre-processed data were segmented from – 1000 to 1000 ms around motor response (i.e., button press). The 500 to 300 ms preceding motor response were used for baseline correction in order to avoid subtracting early ERP activity. Epochs exceeding a voltage threshold of +/−100 µV were excluded from analysis (<0.5%). Because the number of errors committed during in compatible, identical and neutral trials were significantly reduced (see Results for further detail), ERP analyses focused on errors and correct reactions performed in incompatible trials. On average 104.93 correct incompatible trials (SD = 7.29; M_competition_ = 103.73, SD = 8.53, M_cooperation_ = 106.08, SD = 5.76) and 15.48 incorrect incompatible trials (SD = 10.15; M_competition_ = 15.81, SD = 10.67, M_cooperation_ = 15.18, SD = 9.76) were included in ERP analyses. A negative deflection of the ERP is typically elicited after both errors (ERN) and correct reactions (CRN), analyses were thus performed on the difference wave contrasting ERN and CRN – the ∆ERN, isolating activity unique to error-processing. Additional analyses were carried out on the ERN as well as on the CRN. Statistical analyses focused on amplitude values recorded from 0 to 100 ms after motor response at the electrodes FCz and Cz (where the ∆ERN was strongest). The time window for statistical analyses was selected on the basis of previous research (see for instance^69^) and Global Field Power (GFP) analysis. For this purpose, we calculated the standard deviation of ∆ERN amplitude values across all 64 EEG-channels for any given time point of the epoch −1000 to 1000 ms relative to motor response. The resulting time series of spatial standard deviation values, the GFP, resembles an ERP-wave, with peaks indicating the time points associated with the greatest variability across channels (see Supplementary Fig. S1).

### Statistical analyses

All statistical analyses were carried out in the R programming environment^70^. The effects of Social Context, Agency and Affiliation on behavioural and physiological data were assessed via linear (fitted by Maximum Likelihood) mixed-effects regression (using the lme4 package^71^). Similar to ordinary regression, mixed-effects regression (MER) models allow to determine relationships between a set of predictors and response variables (fixed effects). In addition, MER models enable the modelling of individual differences in the response variable’s variance (random effects), which otherwise would increase the error term of ordinary regression, diminishing its reliability and statistical power. Further, a MER approach allowed us to model interdependencies among measurements and handle unbalanced data structures more efficiently, for instance, by nesting data within participants.

Prior to analyses, all categorical variables were effect coded as follows: The Social Context factor (between-subjects; 2 levels: competition vs. cooperation) was effect-coded, such that competition was coded with “1” and cooperation with “−1”. The factor Reaction (within-subjects; 2 levels: error vs. correct) was effect coded such that error was coded with “1” and correct with “−1”. The factor Trial Type (within-subjects; 4 levels: compatible, incompatible, identical, and neutral) described the effect of flanker congruency and was effect-coded, such that neutral trials were coded with “−1”, resulting in three effect coded contrasts: (1) compatible, (2) incompatible, and (3) identical. Participants’ scores in the personality facets Affiliation and Agency, as well as the ∆Engagement difference score were grand-mean centred around zero. All in text reported p-values corrected for multiple comparisons using the Bonferroni method.

## Supplementary information

Supplementary Materials

## Data Availability

Analysis scripts to reproduce the reported results are available through the first author’s GitHub account (https://github.com/JoseAlanis/Supplementary_Soc_ERN). The corresponding datasets are available on reasonable request.
