# Supplementary material for: Social context effects on error-related brain activity are dependent on interpersonal and achievement-related traits
Source: Sci Rep. 2019 Feb 11;9:1728. doi: 10.1038/s41598-018-38417-2 (PMC6370841; doi:10.1038/s41598-018-38417-2)
Supplement: Supplementary file 1 — Supplementary Materials [file 41598_2018_38417_MOESM1_ESM.docx]

# Supplementary Materials

Social context effects on error-related brain activity are dependent on interpersonal and achievement-related traits.

José C. García Alanis^1^, Travis E. Baker^2^, Martin Peper^1^, and Mira-Lynn Chavanon^1, 3^

^1^ Department of Psychology, Experimental and Biological Psychology,
Neuropsychology Section, Philipps-Universität Marburg

^2^ Center for Molecular and Behavioral Neuroscience,
Rutgers University

^3^ Department of Psychology, Child and Adolescent Psychology,
Philipps-Universität Marburg

## Supplementary Methods

| Table S1. Sample Characteristics. | | |
| --- | --- | --- |
|  | M (range) |  |
| **Age** | 23.32 (18, 32) |  |
| **Agency** | 37.46 (-10, 66) |  |
| **Affiliation** | 2.76 (-9, 9) |  |
| **∆Engagement** | 1.55 (- 2.5, 7) |  |
| Note. M = grand mean. | | |

### Correction of ocular and muscle artefacts

EEG data were individually decomposed using adaptive mixture independent component analysis (AMICA ^1,2^). AMICA is an asymptotic newton algorithm that extends the mixture model (cf. ^3^) and Infomax ICA approach (cf. ^4^). It consists of a blind-source separation of the pre-processed EEG data in a Generalized Gaussian mixture model of temporally nearly independent components. This procedure resulted in 64 independent components for each participant. The scalp projection map of each component is believed to represent the relative weight with which a spatially fixed source affects the activity recorded at each electrode ^5^. Here, we fitted a three-shell boundary element head model was to compute the single best-fitting equivalent current dipole that matched the scalp-projection of each component using the DIPFIT toolbox ^6^. The 10-20 electrode locations where aligned with a standard MNI brain model. For correction of stereotypical artefacts, such as eye-movements and muscle activity, we removed independent components for which the equivalent dipole was located outside the brain and explained less than 85% of the variance of the corresponding scalp projection map (cf .^7^). The remaining components where then back projected.

### Statistical Analyses

Analyses were carried out in the R programming environment (R Development Core Team, 2016). Experimental and personality effects on behavioural and physiological data were assessed via linear and generalised linear mixed-effects regression using package lme4 ^8^

To ascertain which model best fitted the data, model fitting was carried out iteratively by (1) fitting an initial 2-level MER model (fitted by Maximum Likelihood) that estimated a random intercept for each participant and controlled for changes in participants’ motivation (i.e., ∆Motivation score). (2) Data-points with excessive influence on the regression (outliers 2.5 standard deviations above and below the residuals’ mean) were removed and the model refitted to the trimmed data. (3) The model was back fitted by removing interaction terms with low relevance (i.e., low F or Chi-square value) and refitting the model. The more complex and simpler models were then compared on the basis of their Akaike’s Information Criterion (AIC); if the interaction terms under consideration did not improve the model fit (more than five points lower AIC-value ^9^) then they were removed, otherwise kept. This was done for all non-significant interaction terms, from higher to lower order. Simple slopes where tested according to Aiken and West ^10^. Multiple comparisons correction was applied using the Bonferroni method implemented in the package emmeans ^11^. Estimates of variance explained by the models are provided using marginal (R^2^_m_; variance explained by the fixed effects only) and conditional R-squared (R^2^_c_; variance explained by the fixed and random effects; cf. ^12^) implemented in the package MuMIn ^13^. For the linear models we provide estimates of effect sizes using semi-partial R-squared (_p_R^2^; cf. ^14^)

Figures were created using the packages ggplot2 ^15^, viridis ^16^, effects ^17^ and custom functions adapted from the package eegUtils ^18^. Tables were created using the package sjPlot ^19^.

## Supplementary Results

*Supplementary Figures*


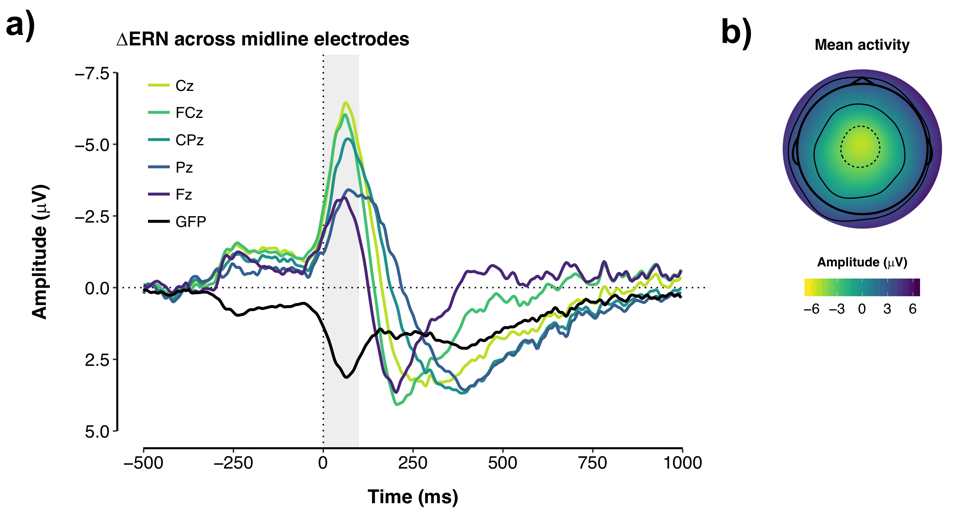


*Supplementary Figure S1*. Grand averaged ∆ERN and global field power.
(**a**) Time course of the ∆ERN across the midline electrodes. (**b**) Topographical map for mean activity recorded from 0 to 100 mis after motor response. As depicted by the Global Field Power (GFP; solid black line in **a**), Activity showed the greatest dissimilarity across electrodes (i.e., more positive GFP-values) from 0 to 100 ms. The strongest (i.e., more negative) ∆ERNs were measured at the electrodes FCz and Cz (b_FCz_ = -4.76, CI = -5.34 – -4.18; b_Cz_ = -5.03, CI = -5.61 – -4.45).

*Supplementary Tables*

| Table S2: Results of linear mixed effects regression analysis of error rates. | | | | |
| --- | --- | --- | --- | --- |
|  | **Full model** | | **Final model** | |
| *Predictors* | *Estimates* | *p* | *Estimates* | *p* |
| (Intercept) | 0.06 (0.05 – 0.07) | **<0.001** | 0.06 (0.05 – 0.07) | **<0.001** |
| Engagement | 0.00 (-0.00 – 0.01) | 0.370 |  |  |
| Compatible (C) | -0.04 (-0.04 – -0.03) | **<0.001** | -0.03 (-0.04 – -0.03) | **<0.001** |
| Identical (I) | -0.03 (-0.04 – -0.02) | **<0.001** | -0.03 (-0.04 – -0.02) | **<0.001** |
| Incompatible (In) | 0.06 (0.06 – 0.07) | **<0.001** | 0.06 (0.05 – 0.07) | **<0.001** |
| Competition | -0.00 (-0.01 – 0.01) | 0.779 | -0.00 (-0.01 – 0.01) | 0.702 |
| C x Competition | 0.00 (-0.01 – 0.01) | 0.557 |  |  |
| I x Competition | -0.00 (-0.01 – 0.01) | 0.507 |  |  |
| In x Competition | 0.00 (-0.01 – 0.01) | 0.561 |  |  |
| **Random Effects** | | | | |
| σ^2^ | 0.00 | | 0.00 | |
| τ_00_ | 0.00 _id_ | | 0.00 _id_ | |
| ICC | 0.48 _id_ | | 0.50 _id_ | |
| Observations | 221 | | 220 | |
| Marginal R^2^ / Conditional R^2^ | 0.386 / 0.682 | | 0.379 / 0.688 | |
| AIC | -712.056 | | -726.308 | |
| Note. Trial type reference level = Neutral. Social context reference level = Cooperation. 95 % confidence interval shown within parenthesis. | | | | |

| Table S3: Results of linear regression analysis of error rates on incompatible trials. | | | | |
| --- | --- | --- | --- | --- |
|  | **Full model** | | **Final model** | |
| *Predictors* | *Estimates* | *p* | *Estimates* | *p* |
| (Intercept) | 0.14 (0.11 – 0.16) | **<0.001** | 0.13 (0.11 – 0.16) | **<0.001** |
| Engagement | 0.01 (-0.00 – 0.02) | 0.193 |  |  |
| Competition | -0.01 (-0.05 – 0.03) | 0.607 | -0.01 (-0.04 – 0.03) | 0.704 |
| Affiliation | 0.01 (0.00 – 0.02) | **0.013** | 0.01 (0.00 – 0.02) | **0.013** |
| Agency | -0.00 (-0.00 – 0.00) | 0.423 | -0.00 (-0.00 – 0.00) | 0.486 |
| Competition x affiliation | -0.01 (-0.02 – -0.00) | **0.034** | -0.01 (-0.02 – -0.00) | **0.044** |
| Competition x agency | 0.00 (-0.00 – 0.00) | 0.272 | 0.00 (-0.00 – 0.00) | 0.370 |
| Observations | 74 | | 74 | |
| R^2^ / adjusted R^2^ | 0.121 / 0.043 | | 0.099 / 0.033 | |
| AIC | -158.709 | | -158.819 | |
| Note. Trial type reference level = Neutral. Social context reference level = Cooperation. 95 % confidence interval shown within parenthesis. | | | | |

| Table S4: Results of linear mixed-effects regression analysis of correct reactions RT. | | |
| --- | --- | --- |
|  | **Final model** | |
| *Predictors* | *Estimates* | *p* |
| (Intercept) | 312.47 (305.94 – 319.00) | **<0.001** |
| Overall error rate | -466.29 (-602.97 – -329.61) | **<0.001** |
| Engagement | 4.55 (0.87 – 8.22) | **0.018** |
| Competition | -2.56 (-9.13 – 4.01) | 0.448 |
| Compatible | -11.16 (-12.90 – -9.42) | **<0.001** |
| Indetical | -11.25 (-12.99 – -9.51) | **<0.001** |
| Incompatible | 17.95 (16.22 – 19.69) | **<0.001** |
| Affiliation | -1.02 (-2.90 – 0.86) | 0.293 |
| Agency | 0.46 (-0.06 – 0.98) | 0.084 |
| **Random Effects** | | |
| σ^2^ | 78.44 | |
| τ_00_ _id_ | 822.98 | |
| ICC _id_ | 0.91 | |
| Observations | 300 | |
| Marginal R^2^ / Conditional R^2^ | 0.444 / 0.952 | |
| AIC | 2466.618 | |
| Note. Trial type reference level = Neutral. Social context reference level = Cooperation. 95 % confidence interval shown within parenthesis. | | |

| Table S5: Results of linear mixed effects regression analysis of delta-ERN by social context. | | | | |
| --- | --- | --- | --- | --- |
|  | **Full model** | | **Final model** | |
| *Predictors* | *Estimates* | *p* | *Estimates* | *p* |
| (Intercept) | -4.96 (-5.54 – -4.37) | **<0.001** | -4.96 (-5.56 – -4.35) | **<0.001** |
| Error rate | 4.83 (-1.90 – 11.56) | 0.164 |  |  |
| Engagement | 0.30 (-0.04 – 0.63) | 0.085 |  |  |
| Competition | -0.67 (-1.26 – -0.08) | **0.028** | -0.70 (-1.31 – -0.09) | **0.027** |
| Affiliation (aff) | -0.27 (-0.45 – -0.10) | **0.003** | -0.24 (-0.42 – -0.06) | **0.010** |
| Agency (ag) | 0.02 (-0.03 – 0.06) | 0.503 | 0.01 (-0.03 – 0.06) | 0.565 |
| Competition x aff | -0.16 (-0.33 – 0.02) | 0.083 | -0.15 (-0.33 – 0.03) | 0.098 |
| Competititon x ag | 0.06 (0.01 – 0.11) | **0.017** | 0.06 (0.02 – 0.11) | **0.012** |
| **Random Effects** | | | | |
| σ^2^ | 4.52 | | 4.51 | |
| τ_00_ | 6.64 _id_ | | 7.17 _id_ | |
| ICC | 0.59 _id_ | | 0.61 _id_ | |
| Observations | 3859 | | 3858 | |
| Marginal R^2^ / Conditional R^2^ | 0.160 / 0.660 | | 0.122 / 0.661 | |
| AIC | 17121.390 | | 17111.043 | |
| Note. Social context reference level = Cooperation. 95 % confidence interval shown within parenthesis. | | | | |

| Table S6: Results of linear mixed effects regression analysis of ERN and CRN. | | |
| --- | --- | --- |
|  | **Final model** | |
| *Predictors* | *Estimates* | *p* |
| (Intercept) | 0.52 (-0.10 – 1.13) | 0.107 |
| Error rate | 7.30 (0.17 – 14.43) | **0.048** |
| Engagement | -0.58 (-0.93 – -0.23) | **0.002** |
| Erroneous reaction (error) | -2.48 (-2.78 – -2.18) | **<0.001** |
| Competition (comp) | -0.32 (-0.94 – 0.31) | 0.322 |
| Affiliation (aff) | -0.07 (-0.25 – 0.12) | 0.478 |
| Agency (ag) | -0.01 (-0.06 – 0.04) | 0.588 |
| Error x comp | -0.37 (-0.67 – -0.06) | **0.020** |
| Error x aff | -0.11 (-0.19 – -0.02) | **0.021** |
| Comp x aff | -0.06 (-0.25 – 0.12) | 0.509 |
| Error x ag | 0.00 (-0.02 – 0.03) | 0.848 |
| Comp x ag | 0.02 (-0.03 – 0.07) | 0.401 |
| Error x comp x aff | -0.08 (-0.17 – 0.01) | 0.081 |
| Error x comp x ag | 0.03 (0.01 – 0.06) | **0.008** |
| **Random Effects** | | |
| σ^2^ | 7.24 | |
| τ_00_ _reaction:id_ | 3.44 | |
| τ_00_ _id_ | 5.77 | |
| ICC _reaction:id_ | 0.21 | |
| ICC _id_ | 0.35 | |
| Observations | 7784 | |
| Marginal R^2^ / Conditional R^2^ | 0.327 / 0.704 | |
| AIC | 38134.526 | |
| Note. Social context reference level = Cooperation. Reaction reference level = correct response. 95 % confidence interval shown within parenthesis. | | |

### Amplitude of the ERN and subsequent behavioural adjustments.

We analysed the functional relationship between the magnitude of the brain’s response to errors and subsequent behaviour adaption. For this purpose, we calculated slowing of RT on trials following error commission (post-error slowing) by subtracting the mean RT for correct reactions that were preceded by correct reactions from mean RT for correct reactions that were preceded by an error. We then fitted a 2-level mixed-effects regression model that estimated a random intercept for each participant and tested how post error slowing was predicted by error-related brain activity. Here, post-error slowing was modelled as a function of ERN amplitude (in preceding error trials), Social Context and the personality facets Affiliation and Agency. In addition, the factor Trial Type was introduced to control for the effect of flanker congruency on post-error trials. Results revealed a significant main effect of ERN amplitude on subsequent post-error slowing (b = -1.56, CI = -2.60– -0.51, p = .004). This indicated that, on average, enhanced (i.e., more negative) ERN amplitudes were associated with a pronounced slowing of RT in consecutive trials (see **Fig. S2**).


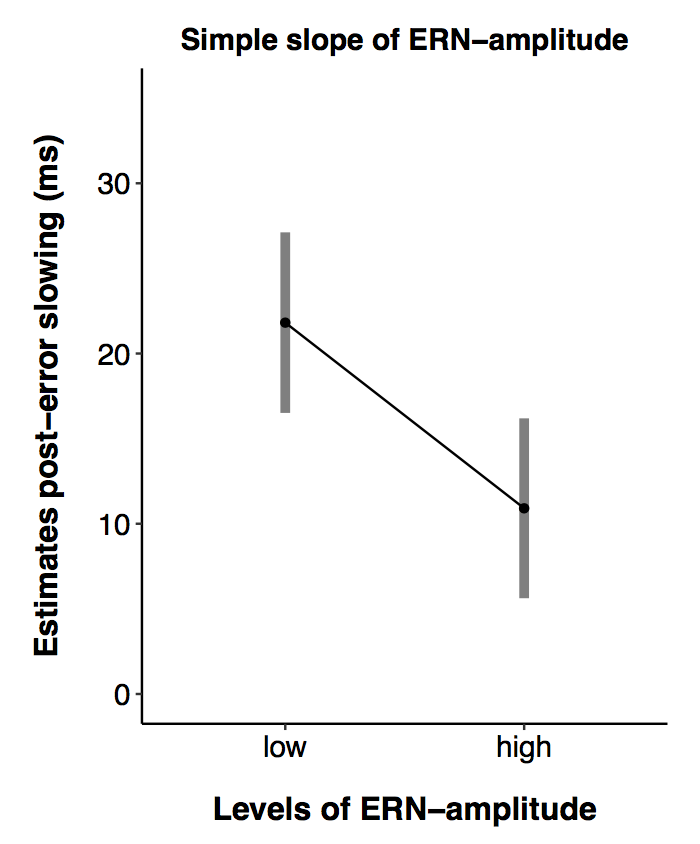


*Supplementary Figure S2.* Post-error slowing as a function of ERN amplitude (previous error).
Predicted values and confidence intervals (95 %) for post-error slowing based on low ERN (- 1SD from overall mean) and high ERN (+ 1 SD) amplitudes in the preceding trial.

Neither affiliation (b = -0.46, CI = -1.52 – 0.61, p = .395) nor agency (b = 0.12, CI = -0.16 – 0.41, p = .380) significantly influenced the strength of post-error slowing. However, we found a significant main effect of social context (b _competition-cooperation_ = -5.40, CI = -12.79 – 2.00, p = .123). As depicted in **Figure S3**, individuals in the cooperation group showed enhanced post-error slowing compared to the competition group.


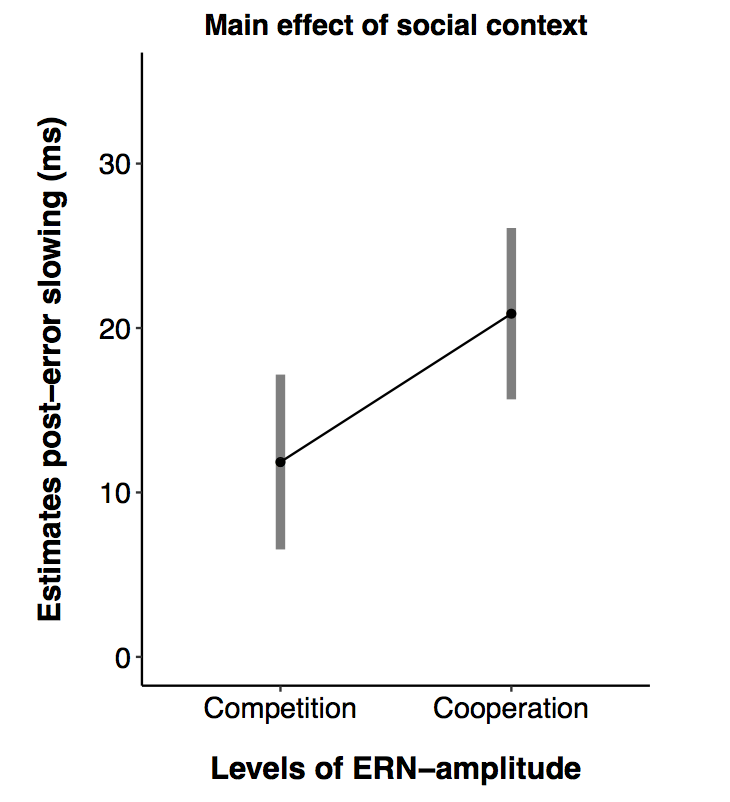


*Supplementary Figure S3.* Post-error slowing as a function of social context
Predicted values and confidence intervals (95 %) for post-error slowing based on social context.

Furthermore, we found a trend for a significant interaction between Social Context and Affiliation (F(1, 272) = 2.93, p = .09.). As depicted in **Fig. S4**, simple slopes indicated that, in the competitive context, high Affiliation individuals showed a more pronounced post-error slowing compared to low affiliation individuals (b _low – high aff. =_ -6.85_,_ CI = 19.81 – 6.14, p = .297). Conversely, in the cooperative context, high Affiliation individuals showed a less pronounced post-error slowing compared to low affiliation individuals (b _high – low ERN =_ 7.32_,_ CI = -4.48 – 18.69, p = .204).


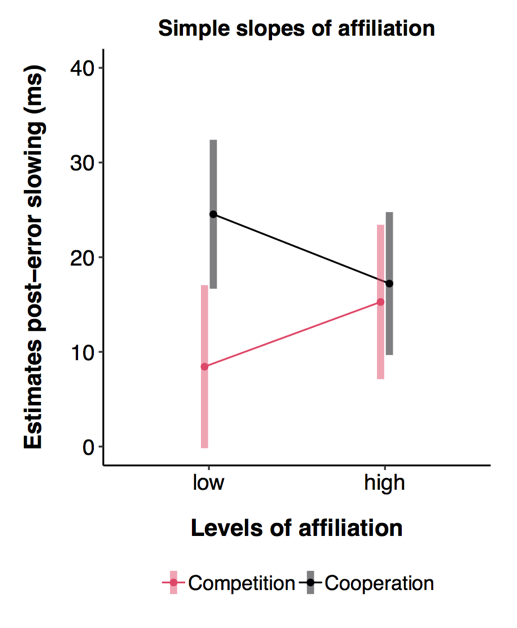


*Supplementary Figure S4.* Combined effects of Affiliation and Social Context on post-error slowing.
Predicted values and confidence intervals (95 %) for post-error slowing based on low (- 1SD from overall mean) and high Affiliation (+ 1 SD) scores.

# References

1. Palmer, J. A., Kreutz-Delgado, K. & Makeig, S. *AMICA: An Adaptive Mixture of Independent Component Analyzers with Shared Components*. (Swartz Center for Computatonal Neursoscience, University of California San Diego., 2011).

2. Palmer, J. A., Makeig, S., Kreutz-Delgado, K. & Rao, B. D. Newton method for the ICA mixture model. in *2008 IEEE International Conference on Acoustics, Speech and Signal Processing* 1805–1808 (2008). doi:10.1109/ICASSP.2008.4517982

3. Lee, T.-W., Lewicki, M. S. & Sejnowski, T. J. ICA mixture models for unsupervised classification of non-Gaussian classes and automatic context switching in blind signal separation. *IEEE Transactions on Pattern Analysis and Machine Intelligence* **22,** 1078–1089 (2000).

4. Makeig, S., Bell, A. J., Jung, T.-P. & Sejnowski, T. J. Independent Component Analysis of Electroencephalographic Data. in *Advances in Neural Information Processing Systems 8* 145–151 (MIT Press, 1996).

5. Onton, J., Westerfield, M., Townsend, J. & Makeig, S. Imaging human EEG dynamics using independent component analysis. *Neuroscience & Biobehavioral Reviews* **30,** 808–822 (2006).

6. Delorme, A. & Makeig, S. EEGLAB: an open source toolbox for analysis of single-trial EEG dynamics including independent component analysis. *Journal of Neuroscience Methods* **134,** 9–21 (2004).

7. Wagner, J., Wessel, J. R., Ghahremani, A. & Aron, A. R. Establishing a Right Frontal Beta Signature for Stopping Action in Scalp EEG: Implications for Testing Inhibitory Control in Other Task Contexts. *Journal of Cognitive Neuroscience* **30,** 107–118 (2018).

8. Bates, D., Mächler, M., Bolker, B. & Walker, S. Fitting Linear Mixed-Effects Models Using lme4. *Journal of Statistical Software* **67,** (2015).

9. Symonds, M. R. E. & Moussalli, A. A brief guide to model selection, multimodel inference and model averaging in behavioural ecology using Akaike’s information criterion. *Behavioral Ecology and Sociobiology* **65,** 13–21 (2011).

10. Aiken, L. S. & West, S. G. *Multiple Regression: Testing and Interpreting Interactions*. (SAGE, 1991).

11. Lenth, R., Love, J. & Herve, M. *emmeans: Estimated Marginal Means, aka Least-Squares Means*. (2018).

12. Nakagawa, S. & Schielzeth, H. A General and Simple Method for Obtaining R2 from Generalized Linear Mixed-Effects Models. *Methods in Ecology and Evolution* **4,** 133–142 (2013).

13. Bartoń, K. *MuMIn: Multi-Model Inference*. (2018).

14. Edwards, L. J., Muller, K. E., Wolfinger, R. D., Qaqish, B. F. & Schabenberger, O. An R2 statistic for fixed effects in the linear mixed model. *Stat Med* **27,** 6137–6157 (2008).

15. Wickham, H. *ggplot2: Elegant Graphics for Data Analysis*. (Springer, 2016).

16. Garnier, S. *viridis: Default Color Maps from ‘matplotlib’.* (2018).

17. Fox, J. Effect Displays in R for Generalised Linear Models. *Journal of Statistical Software* **8,** 27 (2003).

18. Craddock, M. *eegUtils: A collection of utilities for EEG analysis*. (2018).

19. Lüdecke, D. & Schwemmer, C. *sjPlot: Data Visualization for Statistics in Social Science*. (2018).
